# Circulating tumor DNA methylation and gut microbiota in colorectal cancer: diagnostic, prognostic, and therapeutic implications

**DOI:** 10.1007/s10147-026-02966-8

**Published:** 2026-01-24

**Authors:** Kazuhito Sasaki, Shigenobu Emoto, Yuichiro Yokoyama, Soichiro Ishihara

**Affiliations:** Department of Surgical Oncology, Faculty of Medicine, The University of Tokyo, 7-3-1 Hongo, Bunkyo-ku, Tokyo, 113-8655 Japan

**Keywords:** Colorectal cancer, Circulating tumor DNA, DNA methylation, Gut microbiota

## Abstract

Colorectal cancer remains one of the leading causes of cancer-related mortality worldwide, and early detection is essential for improving outcomes. Advances in liquid biopsy technologies and microbiota research have shed new light on diagnostic and therapeutic strategies for colorectal cancer. Notably, circulating tumor DNA methylation has emerged as a sensitive and specific biomarker for early detection, recurrence surveillance, and treatment monitoring. Recent progress in methylation-based assays, including stool- and plasma-derived approaches, highlights their potential clinical utility; however, challenges remain in detecting minimal residual disease at the earliest stages. Parallel to these developments, the gut microbiota has been recognized as a critical modulator of colorectal carcinogenesis and treatment response. Specific bacterial species, such as *Fusobacterium nucleatum*, polyketide synthase-positive *Escherichia coli*, and enterotoxigenic *Bacteroides fragilis*, have been implicated in tumor initiation and progression through epigenetic reprogramming, including aberrant DNA methylation. Microbial metabolites, particularly short-chain fatty acids such as butyrate, influence DNA methyltransferase activity and histone modifications, linking microbial ecology to the host epigenome. Microbiota composition also affects responses to chemotherapy, radiotherapy, and immunotherapy, underscoring its potential as a predictive biomarker and therapeutic target. Integrating circulating tumor DNA methylation profiling with microbiota analysis provides a promising strategy for personalized colorectal cancer management, combining early detection with treatment outcome prediction. This review summarizes the current evidence and future directions at the interface of DNA methylation and the gut microbiota, emphasizing their synergistic role as composite biomarkers in precision oncology.

## Introduction

Colorectal cancer (CRC) is the third most common solid malignant tumor worldwide and the third leading cause of cancer-related mortality [1]. Its long progression allows precancerous lesions (adenomas) to develop into malignant lesions, enabling early CRC detection through screening [2]. Several methods are currently available for detecting CRC, including fecal immunochemical testing (FIT), fecal occult blood testing, and colonoscopy. Despite advances in antitumor drug research, the efficacy of CRC treatment remains limited due to late-stage diagnosis and inadequate patient selection. Although colonoscopy is widely used, low patient compliance among the general population due to burdensome bowel preparation and potential complications limits its application. These issues underscore the need for novel diagnostic methods or effective biomarkers to enable early detection and guide personalized treatment.

**Fig. 1:**
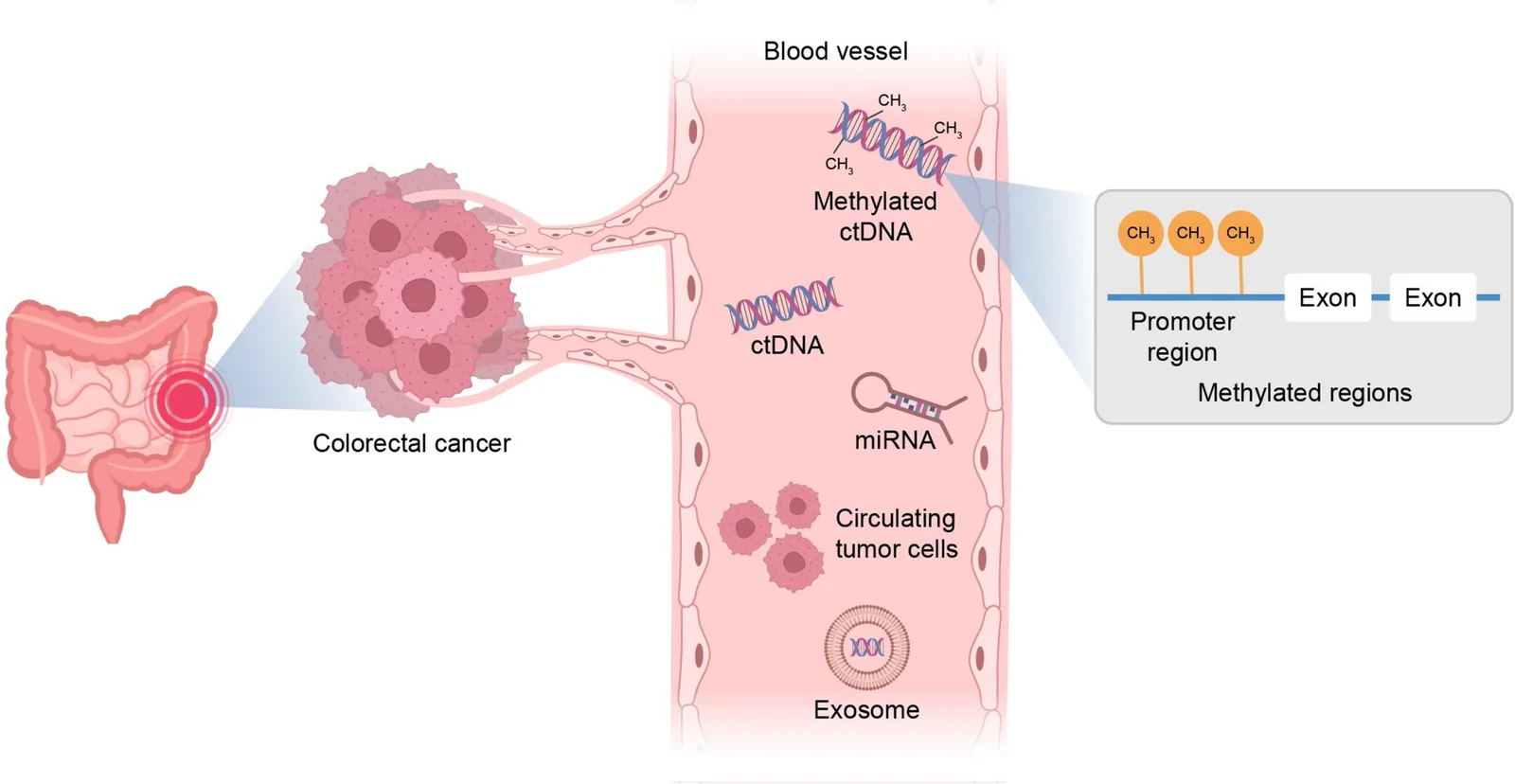
Sources of circulating tumor DNA in body fluids. Mode of circulating tumor DNA (ctDNA) entry into the bloodstream. The ctDNA in the bloodstream originates from circulating tumor cells, exosomes secreted by tumor cells, and apoptotic and necrotic tumor cells, all of which contain methylated ctDNA

**Fig. 2:**
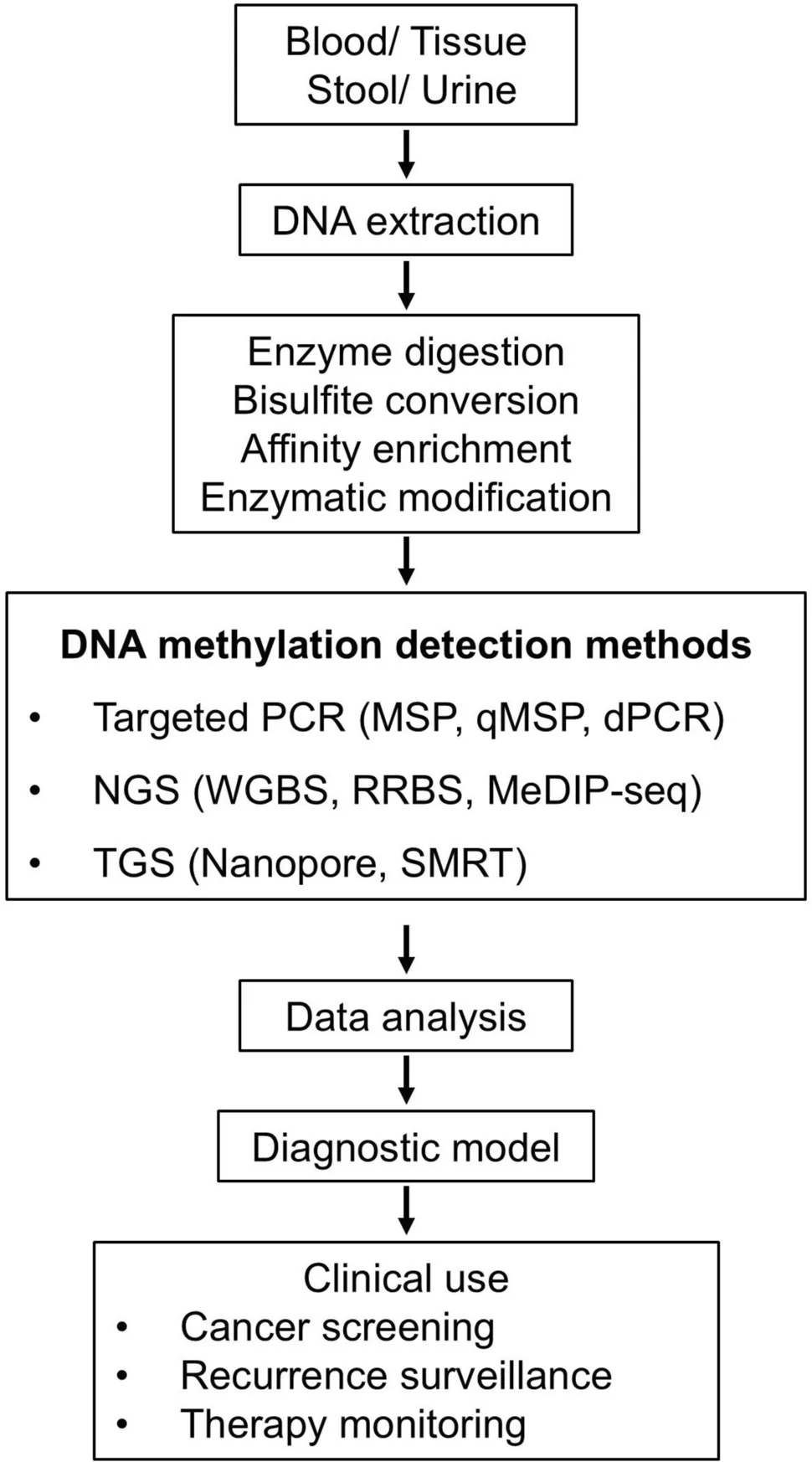
Workflow diagram for the clinical use of methylation biomarkers. Circulating tumor DNA methylation assays for CRC include bisulfite-based methods, targeted methylation NGS panels, whole-genome bisulfite sequencing (WGBS), bisulfite-free enrichment, or enzymatic conversion strategies, such as cell-free methylated DNA immunoprecipitation sequencing (MeDIP-seq), third-generation long-read sequencing (TGS), including nanopore sequencing, and single-molecule real-time (SMRT) sequencing. Bisulfite conversion remains a reference standard for methylation-specific PCR (MSP) and quantitative methylation-specific PCR (qMSP)/digital PCR (dPCR) due to its high analytical sensitivity for locus-specific detection

Significant advances have been made in the investigation of liquid biopsies and gut microbiota across various cancer types. A liquid biopsy analyzes specimens obtained from blood or other body fluids, targeting components such as circulating tumor cells, circulating tumor DNA (ctDNA), exosomes, and microRNAs. Among these, ctDNA are cell-free DNA (cfDNA) fragments released from tumor cells into the peripheral blood [3] (Fig. 1). They originate from tumor cell necrosis or apoptosis and carry tumor-specific genetic and epigenetic information [3]. ctDNA can serve as an alternative source of tumor DNA for tumor diagnosis and prognosis [4–7] (Fig. 2). DNA methylation is a major epigenetic modification that is closely associated with the occurrence and development of malignant epithelial tumors. Aberrant methylation is a primary feature of early tumorigenesis, characterized by changes in DNA methylation status and gene expression disruptions [5, 6, 8, 9]. Different methylation-based assays vary in their sensitivity and clinical utility. Methylation-specific PCR provides a rapid and cost-effective approach, but is limited by its qualitative nature and dependence on predefined CpG sites [10]. Targeted bisulfite sequencing and digital PCR enable more quantitative detection and improve performance when tumor fractions are low [11]. Cell-free methylated DNA immunoprecipitation sequencing (cfMeDIP-seq) offers a bisulfite-free, low-input strategy suitable for early detection [10]. Pre-analytical factors, including leukocyte-derived cfDNA background and variability in the tumor fraction, remain key determinants influencing assay sensitivity [11].

The development of next-generation sequencing technology in the early twenty-first century has greatly accelerated research on the human gut microbiota. Dysbiosis has been linked to the onset and progression of various chronic diseases, including cancer, diabetes, and inflammatory bowel disease [12–14]. Considerable attention has been focused on the relationship between specific bacterial species and tumorigenesis in CRC. Some bacteria, including *Fusobacterium nucleatum*, polyketide synthase-positive *Escherichia coli*, and enterotoxigenic *Bacteroides fragilis*, are reported to promote tumor development [12, 15]. Therefore, microbial profiling of the gut flora is increasingly recognized for its potential utility in cancer prevention and early detection. Recent studies show that the gut microbiota help modulate responses to cancer treatments, including chemotherapy, immunotherapy, and radiotherapy [12]. Microbial metabolites may influence drug metabolism, thereby enhancing or attenuating the therapeutic efficacy of drugs [16]. The efficacy of immune checkpoint inhibitors (ICIs) partly depends on gut microbiota diversity and composition [17]. These findings highlight the gut microbiota as a key determinant of cancer treatment outcomes and not merely a component of the intestinal environment.

This review summarizes the current applications of ctDNA methylation and gut microbiota in CRC.

## Clinical utility of ctDNA methylation for early detection, recurrence monitoring, and treatment response assessment in CRC

### Early detection using stool-based testing

FIT is the most widely implemented method for early CRC detection worldwide. A 2020 review summarized its sensitivity and specificity across different cancer stages [18]. While FIT demonstrates an overall sensitivity exceeding 70%, its sensitivity for stage I CRC is 73%, with a specificity of 89%. The sensitivity for T1 CRC is 40%, raising concerns about its effectiveness in detecting early-stage disease. These findings highlight the need to further improve FIT’s performance for early CRC detection.

A multitarget stool DNA test, developed in 2014 to address these limitations, combines FIT with the detection of stool-based genetic and epigenetic markers, including *KRAS* mutations and *NDRG4* and *BMP3* methylation, along with β-actin as a reference gene [19]. More recently, a next-generation multitarget stool DNA test was reported in 2024, incorporating FIT and *LASS4*, *LRRC4*, *PPP2R5C*, and *ZDHHC1* methylation analyses [20]. The test demonstrated a sensitivity of 93.9% for CRC, with a specificity of 90.6% for detecting advanced neoplasia—including multiple adenomas or large (≥ 1 cm) sessile serrated lesions. Furthermore, the test achieved nearly 90% sensitivity for stage I CRC and 88.2% sensitivity for right-sided advanced neoplasia. Although the sensitivity improved significantly compared to FIT alone, the specificity was lower.

Interest in clinical applications has intensified with epigenetic-based CRC screening solutions. Registered tests employing DNA methylation for CRC screening using fecal samples include Cologuard (Exact Sciences Corporation, Madison, WI, USA), ColoClear (New Horizon Health Technology Co., Ltd., Hangzhou, China), Earlytect Colon (Genomictree, Inc., Daejeon, South Korea), Colosafe (Creative Biosciences Guangzhou Co., Ltd., Guangzhou, China), and Colowell (Shanghai Realbio Technology Co., Ltd., Shanghai, China) [21]. Cologuard and ColoClear analyze the methylation of *NDRG4* and *BMP3*, along with *KRAS* mutations, whereas the other tests rely on *SDC2* methylation status. The National Comprehensive Cancer Network Guideline for CRC Screening (version 2.2025) recommends the use of Cologuard as a CRC screening strategy.

### Early detection using blood-based assays

Liquid biopsy targeting ctDNA in blood samples can be broadly categorized into the detection of somatic mutations and epigenetic changes, including methylation alterations. Mutation-based assays require prior knowledge of the specific mutations in the tumor tissue. While these assays are highly valuable for identifying molecular residual disease (MRD) post-treatment, they are less suitable for distinguishing between healthy individuals and patients with cancer in a screening context.

Consequently, methylation-based assays, which detect epigenomic alterations, are primarily utilized for early cancer detection. The first Food and Drug Administration-approved blood-based methylation assay was Epi proColon (Epigenomics AG, Berlin, Germany/US laboratory partner), which detects Septin9 methylation in plasma samples [22]. However, its clinical performance was suboptimal for population-based screening, with a sensitivity and specificity of 52.6% and 66.7%, respectively, for stage I CRC. Subsequent efforts have focused on expanding the number of methylation markers and assembling panels to enhance diagnostic performance. Despite gradual improvements, most reports indicate that the sensitivity for stage I CRC is approximately 60% when the specificity threshold is set at approximately 95% [23].

In 2021, Klein et al. reported a novel early detection tool that combined a methylation panel with artificial intelligence (AI)-based analysis [24]. This multi-cancer detection method identified ten cancer types—including colorectal, bladder, esophageal, head and neck, liver, bile duct, pancreatic, lymphoid, ovarian, and gastric cancers—from a single blood specimen. Regarding CRC, the test demonstrated a specificity of 95.5%. The sensitivity for stage I CRC was relatively low at 43.3%, while those for stages II, III, and IV were 85.0%, 87.9%, and 95.3%, respectively, indicating a strong performance in more advanced disease stages.

Although early CRC detection is crucial for improving patient prognosis, its identification using ctDNA methylation analysis remains challenging. The major limitation is that early tumors release only small amounts of ctDNA into the circulation, resulting in substantially lower detection sensitivity than in advanced stages [11]. Contamination with normal hematopoietic cell-derived cfDNA makes it technically challenging to distinguish real tumor-derived methylation signals. DNA methylation detection has advanced beyond methylation-specific polymerase chain reaction (PCR) and enzyme assays, with digital PCR, ultra-deep sequencing, cell-free methylated DNA immunoprecipitation sequencing, and AI-based profiling, enabling more sensitive detection of ctDNA, potentially overcoming current limitations in stage I CRC [10].

### Liquid biopsy utility for recurrence surveillance and therapeutic monitoring

Numerous studies have demonstrated the utility of ctDNA in monitoring postoperative recurrence and identifying patients at high risk of relapse [25]. ctDNA can be detected in blood samples several months before recurrent disease becomes radiologically apparent.

Historically, multigene panels targeting tumor-specific somatic mutations have been used for ctDNA-based detection in various solid tumors. However, these panels may not include all mutations present in an individual patient’s tumor, potentially limiting sensitivity and leading to false-negative results. A personalized, tumor-informed approach has recently been developed to address this limitation. This method involves generating a personalized multigene mutation panel based on the genetic profile of each patient’s resected tumor tissue, enabling highly sensitive and individualized detection of ctDNA [10, 26, 27]. A 2023 study from Japan applied this approach to patients with CRC and stratified individuals into high- and low-risk groups for postoperative recurrence [28]. In the above-mentioned study, patients with detectable ctDNA 1 month postoperatively had significantly worse 3-year disease-free survival than those without—38.4% versus 90.5%. These striking results underscore the prognostic power of ctDNA detection in the postoperative setting.

However, the tumor-informed approach described above requires a customized panel design for each patient, presenting practical limitations in terms of time and cost, particularly considering the high incidence of CRC. As an alternative, recent efforts have focused on identifying MRD by detecting methylation signatures in ctDNA. Methylation-based assays have several advantages: they do not require prior tumor genotyping or individualized mutation panels, making them more cost-effective and scalable. Recent studies have demonstrated the utility of tumor-naïve ctDNA assays incorporating DNA methylation for predicting recurrence in stage II–III CRC [29, 30]. The UK TRACC Part B study, using the Guardant Reveal assay, demonstrated that postoperative ctDNA detection was strongly associated with reduced 2-year recurrence-free survival, with a high negative predictive value (91.2%) and a median lead time of 7.3 months to radiologic recurrence [29]. Similarly, the CIRCULATE-Japan GALAXY study validated the Tempus xM tumor-naïve ctDNA assay [30]. At 4 weeks postoperatively, the assay achieved 61.1% sensitivity and 87.9% specificity, improving to 83.3% and 89.5%, respectively, with longitudinal monitoring [30].

Key needs include harmonized positivity thresholds, definitions of persistence/clearance, and prospective trials testing ctDNA-triggered interventions. The convergence of plasma-only methylation-integrated evidence with large prospective programs is poised to accelerate the standardization of CRC surveillance and therapeutic monitoring.

## Gut microbiota and prediction of therapeutic response to preoperative chemoradiotherapy in CRC

### Gut microbiota and CRC

The human colon harbors approximately 100 trillion microorganisms representing 1000–1500 bacterial species [31]. The gut microbiota composition is highly diverse and influenced by numerous factors, including ethnicity, sex, age, dietary habits, fetal environment, and medication use [13]. It is known as a “hidden organ” and plays essential physiological roles in immune modulation, metabolic regulation, and protection against infection [13, 32]. Although numerous mechanistic studies have proposed microbe–epigenome interactions in CRC, only a subset of these findings have been validated in well-designed clinical cohorts. Distinguishing exploratory mechanistic insights from clinically established associations is essential for a balanced interpretation of the microbiota–CRC literature.

Dysbiosis refers to any abnormalities in microbiota function and/or composition that are associated with disease pathogenesis, including CRC [33]. *Fusobacterium nucleatum* is frequently enriched in CRC and contributes to tumorigenesis through immune modulation and oncogenic signaling [12–14, 34]. Large-scale cohort studies analyzing the gut microbiota of patients with CRC have revealed an increased abundance of several bacterial species in stool samples [35, 36]. Yachida et al. reported that species including *Atopobium parvulum* and *Actinomyces odontolyticus* were enriched in early lesions such as polypoid adenomas and intramucosal carcinomas [36]. However, the 2024 LCPM study emphasized the importance of confounding variables and showed that some traditional CRC-associated bacteria, including *Fusobacterium nucleatum,* lost statistical significance under strict confounder control, while others, including *Parvimonas micra* and *Peptostreptococcus anaerobius,* remained significant [35]. These findings suggest that fecal microbiota composition could serve as a diagnostic tool for CRC; however, various adjustments remain necessary due to the influence of diet and lifestyle habits.

### Microbiota influence on treatment response and adverse events

Many studies have demonstrated that the gut microbiota influences tumor survival and progression through multiple mechanisms, including direct interactions between bacteria and cancer cells, production of metabolites that modulate tumor growth, and regulation of local and systemic immune responses [12, 37]. The impact of gut microbiota on chemotherapy outcomes in CRC has recently attracted increasing attention. Growing evidence suggests that the gut microbiota can modulate the response of patients with CRC to chemopreventive and chemotherapeutic agents, including aspirin [38], 5-fluorouracil [39, 40], irinotecan [41], and oxaliplatin [16, 42]. Several mechanisms might explain these effects, including microbial biotransformation, chemoresistance pathway modulation, and therapy-elicited immunity. *Prevotella* impairs the antitumor effect of folinic acid, fluorouracil, and oxaliplatin by producing the bile acid metabolite 3-oxocholic acid, which activates the epidermal growth factor receptor/extracellular signal-regulated kinase/c-myelocytoma proto-oncogene signaling pathway and promotes tumor progression [40]. However, short-chain fatty acids (SCFAs), particularly commensal bacteria-derived butyrate, enhance the therapeutic efficacy of oxaliplatin by strengthening CD8⁺ T cell-mediated antitumor immunity. This effect is mediated via ID2-dependent interleukin-12 signaling, and clinical evidence indicates that higher serum butyrate levels are associated with improved responses to oxaliplatin [16].

Gut microbiota manipulation in CRC radiotherapy can enhance sensitivity to treatment and reduce radiation-associated toxicity [43]. A two-way interaction exists between intestinal microbiota and radiotherapy. The intestinal microbiota influences the antitumor efficacy of radiotherapy, while ionizing radiation alters its composition and function, which consequently contributes to the development of radiation enteropathy [44, 45]. Therefore, the intestinal microbiota is of considerable interest in radiotherapy, both as a potential protective agent and as a biomarker of radiation exposure. A clinical study demonstrated that pelvic radiotherapy remodeled the overall gut microbiota composition, including a 10% decrease in *Firmicutes* and a 3% increase in *Fusobacterium* [46]. Another study analyzing 45 fecal samples from patients with rectal cancer pre-chemoradiotherapy (CRT) found that members of *Bacteroidales*—including *Bacteroidaceae*, *Rikenellaceae*, and *Bacteroides*—were relatively more abundant in patients with a non-complete response (CR) than in those with a CR [47]. *Duodenibacillus massiliensis* was associated with an improved CR rate. The most notable changes in the gut microbiota after cytotoxic chemotherapy or radiotherapy include increases in *Bacteroides* and *Enterobacteriaceae,* along with decreases in *Bifidobacterium*, *Faecalibacterium prausnitzii*, and *Clostridium* cluster XIVa [48].

A prospective study evaluated the association of gut microbiota with CRT efficacy and toxicity in patients with locally advanced lower rectal cancer treated with the tegafur–uracil/calcium folinate/irinotecan regimen [49, 50]. Twenty-one patients were enrolled, and mucosal and stool samples were collected pre- and post-CRT for 16S rRNA sequencing. Microbial α- and β-diversity were assessed, and linear discriminant analysis effect size analysis was used to identify taxa related to treatment response and side effects. Overall diversity showed no significant differences between the pathological CR (pCR) and non-pCR groups or among different grades of diarrhea severity; however, in tumor tissue, β-diversity showed a significant change before and after treatment. Notably, specific bacterial taxa were associated with clinical outcomes. In pre-CRT tumor mucosa, *Peptostreptococcus*, *Coprococcus*, and *Phoceaicola* were associated with pCR, potentially through immune modulation or intratumoral metabolism. *Collinsella*, *Haemophilus*, and *Desulfovibrionaceae* were linked to diarrhea in non-tumor mucosa, likely due to their roles in mucosal inflammation and gut barrier dysfunction [50].

### Gut microbiota and immune checkpoint inhibitors in CRC

Recent advances in immunotherapy have transformed the treatment landscape of CRC [17]. ICIs targeting programmed cell death protein 1 (PD-1), programmed cell death ligand 1, and cytotoxic T lymphocyte-associated protein 4 have shown remarkable efficacy in microsatellite instability-high or mismatch repair (MMR)-deficient CRC [51]. Emerging evidence suggests that the gut microbiota modulate the efficacy and toxicity of ICIs, providing a potential avenue to overcome resistance in non-responders. Regarding antitumor immune response modulation, commensal bacteria, including *Akkermansia muciniphila*, *Bifidobacterium longum*, *Faecalibacterium prausnitzii*, and *Ruminococcaceae,* have been associated with enhanced ICI responsiveness, possibly through the promotion of dendritic cell maturation, activation of CD8 + T cells, and increased production of SCFAs, including butyrate [17]. In CRC, *Fusobacterium nucleatum* may induce myeloid-derived suppressor cells to suppress antitumor immunity, leading to resistance to PD-1 inhibitor treatment [52]. Fecal microbiota transplantation (FMT) enhances the antitumor immune response by introducing healthy microbiota, thereby rebuilding the gut microbiota and restoring a patient’s microbiota diversity and functionality [17, 53]. Collecting feces from ICI-responsive patients and administering FMT significantly improves the immunotherapy response in non-responders [54]. A healthy microbial population enhances T-cell activity, which synergizes with the mechanism of PD-1 inhibitors to strengthen antitumor immune responses [53]. Patients with MMR-proficient (pMMR) CRC have an immunosuppressive tumor microenvironment, limiting the benefits of ICIs. However, combined treatment with FMT improves therapeutic outcomes for such patients, as confirmed by clinical cases of melanoma treatment [55]. Among ten patients, clinical responses were observed in three—two partial responses and one CR. This effect was associated with favorable changes in immune cell infiltrates and gene expression profiles in the tumor microenvironment and gut lamina propria. Cheng et al. reported that FMT improved ICI response in patients with pMMR and microsatellite-stable CRC [56]. Clinical trials examining the exact efficacy of FMT in treating pMMR CRC combined with ICI are still ongoing (NCT04729322 and NCT05279677), with highly anticipated outcomes.

## DNA methylation and the gut microbiota in CRC

**Fig. 3:**
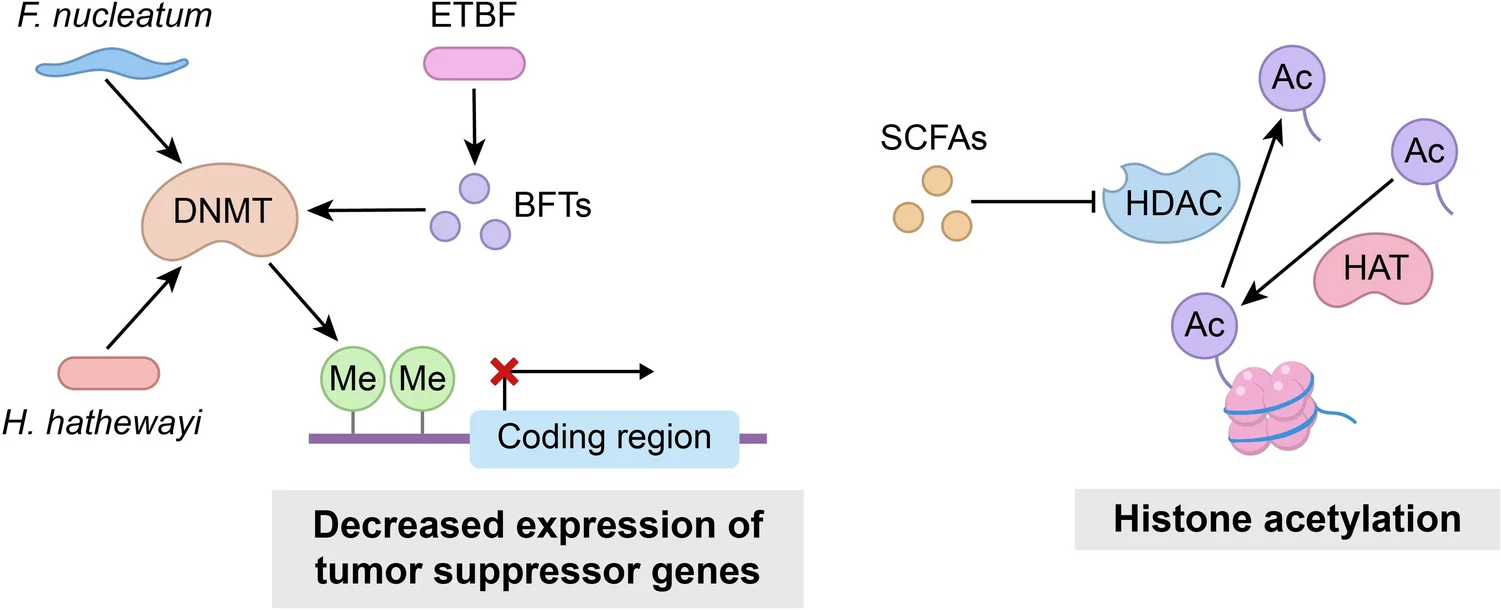
Mechanistic diagram of microbiota-DNA methylation interactions. *Fusobacterium nucleatum and Hungatella hathewayi* induce promoter hypermethylation by dysregulating DNA methyltransferases (DNMTs). *Bacteroides fragilis* toxin (BFT)-induced increase in DNMT activity results in aberrant DNA methylation. Short-chain fatty acids (SCFAs) inhibit histone deacetylase (HDAC), increase histone acetylation, and alter CpG accessibility through open chromatin formation. *Ac* acetyl group, *ETBF* enterotoxigenic *Bacteroides fragilis*, *HAT* histone acetyltransferase

Emerging data indicate that the gut microbiota can program host methylomes in the colorectum, thereby influencing cancer risk and disease biology [57, 58]. Gut microbial influence on host methylation may occur through several pathways (Fig. 3). SCFAs modulate epigenetic states via histone deacetylase inhibition and DNA methyltransferases (DNMT) regulation [59, 60], whereas dysbiosis-related inflammatory signaling can alter chromatin accessibility [10]. Toxin-mediated mechanisms, including chromatin remodeling induced by *Bacteroides fragilis* toxin (BFT), further highlight microbe–epigenome interactions [61]. These mechanistic insights remain preliminary, but may contribute to circulating methylation signatures detected in ctDNA [16]. In an integrative multi-omic analysis of CRC tissues and paired microbiome profiles, specific microbial consortia were found to correlate with locus-specific methylation changes across cancer-relevant pathways, providing evidence that microbes help “write” host methylation patterns during tumor evolution [62].

Few studies have explored the influence of microbiota on gene-specific methylation (Table 1). CRC-enriched species, notably *Fusobacterium nucleatum*, have been consistently linked to methylation-defined tumor subtypes [63, 64]. Tahara et al. reported associations between intratumoral *Fusobacterium* burden and CpG island methylator phenotype (CIMP) positivity, as well as MLH1 promoter methylation [64]. Mechanistically, microbe-derived metabolites and inflammatory signaling provide plausible routes from colonization to methylome remodeling. *Hungatella hathewayi* has also been linked to hypermethylation of tumor-suppressor and differentiation-related genes, potentially promoting CIMP-like epigenetic patterns by upregulating DNMTs [57]. SCFAs modulate chromatin through histone deacetylase inhibitor and broader epigenetic effects, with downstream impacts on cell cycle control and immune tone [59, 60]. BFT-induced chromatin accessibility changes overlap with common differentially methylated regions in CRC, particularly at hypomethylated loci, suggesting that BFT contributes to tumorigenesis by epigenetically remodeling chromatin to facilitate aberrant DNA methylation patterns [61]. Liu et al. performed metagenomic sequencing of fecal samples from matched patients with CRC and healthy controls [62]. Their study found that *Fusobacterium and Peptostreptococcus,* which are enriched in CRC tissues, were associated with DNA methylation changes in cancer cells, particularly in regions regulating tumor suppressor genes, suggesting a microbe-driven epigenetic mechanism contributing to oncogenesis [62].

**Table 1:** Effects of gut microbiota on DNA methylation in colorectal cancer

Gut microbiota	Sample source	Type of evidence	DNA methylation changes	Proposed mechanism	Key references
*Fusobacterium nucleatum*	Tumor tissue	Clinical	CIMP positivity; MLH1 promoter hypermethylation	Induces chronic inflammation; recruits MDSCs;	[63, 64]
SCFA-producing bacteria	Stool, mucosa	Preclinical + clinical	Butyrate inhibits HDAC, indirectly influencing DNA methylation patterns (gene activation of tumor suppressors)	SCFA modulates DNMT activity and acts as an HDAC inhibitor, remodeling chromatin and CpG methylation	[59, 60]
*Enterotoxigenic Bacteroides fragilis*	Mucosa, epithelial cells	Preclinical	Hypermethylation of tumor suppressor genes in colonic epithelial cells	Toxin-induced DNA damage and ROS production enhance DNMT activity, driving aberrant methylation	[61]
*Fusobacterium* + *Peptostreptococcus* (co-enrichment)	Tumor tissue	Clinical	Enrichment correlates with global hypermethylation patterns in CRC tissue	Promote inflammatory cytokines and metabolites influencing methyltransferase activity	[62]
*Hungatella hathewayi*	Tumor tissue	Clinical	Correlated with hypermethylation of multiple tumor-suppressor and differentiation-related genes	Enhance DNMT expression/activity, promoting CIMP within Wnt and epithelial differentiation pathways	[57]

*SCFA* short-chain fatty acid, *CIMP* CpG island methylator phenotype, *HDAC* histone deacetylase, *CRC* colorectal cancer, *MDSC* myeloid-derived suppressor cell, *DNMT* DNA methyltransferase, *ROS* reactive oxygen species

Population-level studies have further linked common gut taxa to differential methylation in host blood and tissue, reinforcing that microbial ecology and host epigenetics are intertwined at scale [65, 66]. Tumor DNA methylation may indirectly reflect gut microbiota interactions, and future strategies are expected to integrate microbiome markers with ctDNA methylation as composite biomarkers.

**Fig. 4:**
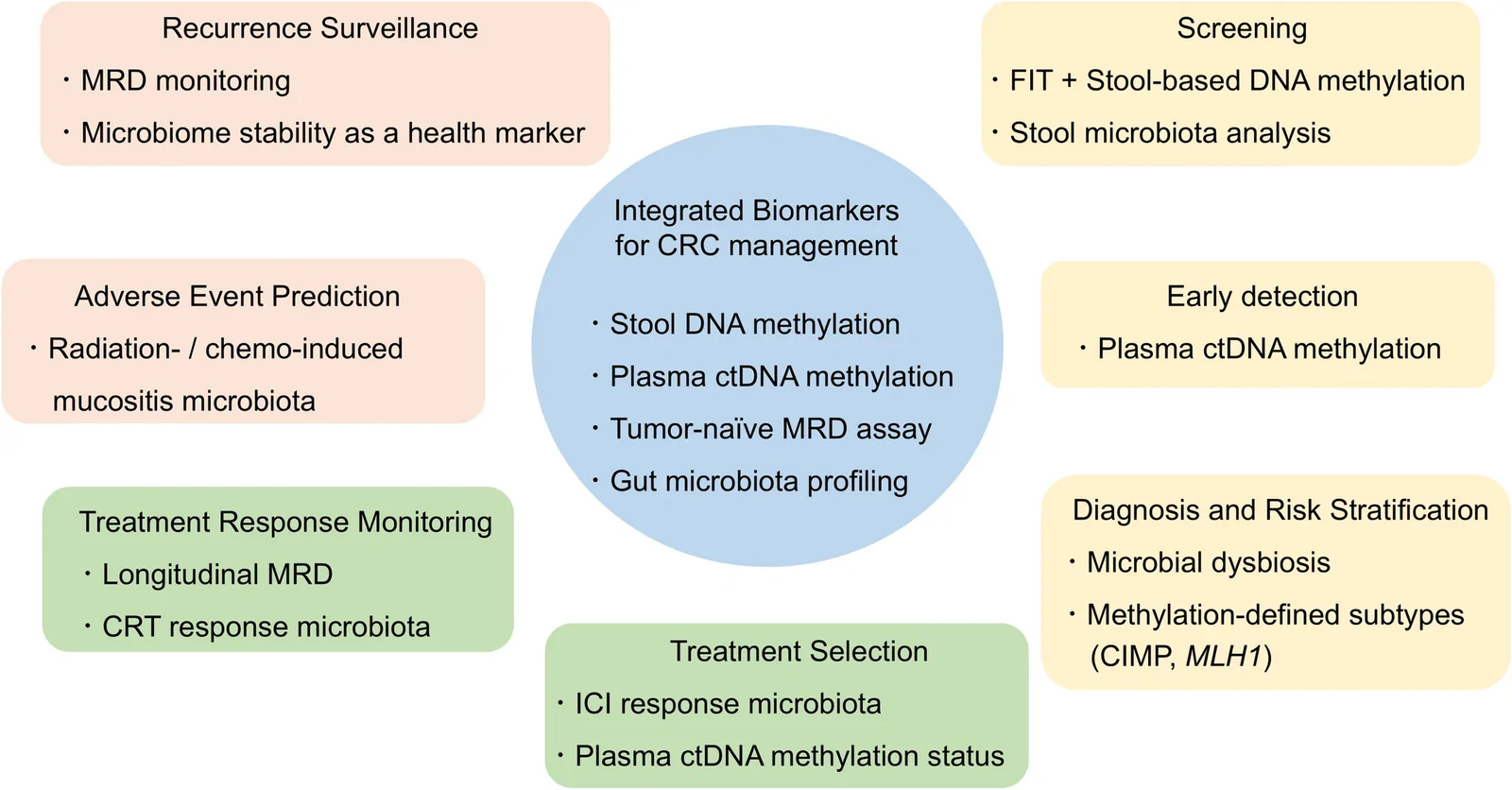
Integrated biomarker framework for colorectal cancer management. This conceptual model summarizes how multi-omics biomarkers, including stool DNA methylation, plasma ctDNA methylation, tumor-naïve ctDNA minimal residual disease assays, and gut microbiota profiling, can be applied across the clinical continuum of colorectal cancer. These biomarkers support screening, early detection, diagnosis, risk stratification, treatment selection, monitoring of treatment response, prediction of treatment-related adverse events, and surveillance for recurrence. The central hub highlights the complementary and potentially synergistic contributions of methylation-based liquid biopsy and microbiota-derived signatures to precision oncology. *CRC* colorectal cancer, *ctDNA* circulating tumor DNA, *FIT* fecal immunochemical testing, *MRD* minimal residual disease, *CIMP* CpG island methylator phenotypz, *ICI* immune checkpoint inhibitor, *CRT* chemoradiotherapy

Figure 4 shows an integrated conceptual model summarizing how multi-omics biomarkers, specifically stool and plasma DNA methylation, tumor-naïve ctDNA assays, and gut microbiota profiling, can be applied across the clinical trajectory of CRC. These biomarkers contribute not only to screening and early detection, but also to risk stratification, treatment selection, assessment of therapeutic response, prediction of treatment-related adverse events, and surveillance for recurrence. This framework highlights the complementary roles of ctDNA methylation and microbiota signatures in precision oncology.

## Conclusion

This review outlines the current status of liquid biopsy and gut microbiota in CRC, including our institutional research findings. Remarkable progress has been made in the use of ctDNA for MRD detection, with the potential to transform CRC care in the near future, if issues such as cost can be resolved. Our research also suggests that the gut microbiota plays a pivotal role in determining treatment response and adverse effect profiles during CRT for rectal cancer. These insights open new avenues for developing microbiota-targeted therapeutic strategies, and ongoing research is expected to further advance this field.

## Data Availability

No new data were generated or analyzed in this study. Therefore, data sharing is not applicable.
